# High pathogen prevalence in an amphibian and reptile assemblage at a site with risk factors for dispersal in Galicia, Spain

**DOI:** 10.1371/journal.pone.0236803

**Published:** 2020-07-30

**Authors:** Marius von Essen, William T. M. Leung, Jaime Bosch, Simon Pooley, Cesar Ayres, Stephen J. Price

**Affiliations:** 1 Institute of Zoology, Zoological Society of London, Regent’s Park, London, United Kingdom; 2 Imperial College London, Department of Life Sciences (Silwood Park), Ascot, United Kingdom; 3 Museo Nacional de Ciencias Naturales, CSIC, Madrid, Spain; 4 Research Unit of Biodiversity—CSIC/UO/PA, Universidad de Oviedo, Edificio de Investigación, Mieres, Spain; 5 Asociación Herpetológica Española, Madrid, Spain; 6 UCL Genetics Institute, London, United Kingdom; University of South Dakota, UNITED STATES

## Abstract

Ranaviruses are agents of disease, mortality and population declines in ectothermic vertebrates and emergences have been repeatedly linked to human activities. Ranaviruses in the common midwife toad ranavirus lineage are emerging in Europe. They are known to be severe multi-host pathogens of amphibians and can also cause disease in reptiles. Recurrent outbreaks of ranavirus disease and mortality affecting three species have occurred at a small reservoir in north-west Spain but no data were available on occurrence of the pathogen in the other amphibian and reptile species present or at adjacent sites. We sampled nine species of amphibians and reptiles at the reservoir and nearby sites and screened for ranavirus presence using molecular methods. Our results show infection with ranavirus in all nine species, including first reports for *Hyla molleri*, *Pelophylax perezi*, *Rana iberica*, and *Podarcis bocagei*. We detected ranavirus in all four local sites and confirmed mass mortality incidents involving *Lissotriton boscai* and *Triturus marmoratus* were ongoing. The reservoir regularly hosts water sports tournaments and the risks of ranavirus dispersal through the translocation of contaminated equipment are discussed.

## 1. Introduction

Globally, emerging infectious diseases (EIDs) are receiving considerable attention as a driver of amphibian declines [1–3]. The World Organisation for Animal Health (OIE) has listed ranavirosis as an internationally notifiable disease of amphibians [4,5]. Members of the genus *Ranavirus* (family *Iridoviridae*) are important amphibian pathogens, that also cause disease in reptiles, and fish [6–9].

The known distribution of ranaviruses in Europe remains patchy but several lineages have been isolated [10–12]. It is not clear how long ranaviruses have been associated with ectothermic vertebrates in Europe but they are thought to have been introduced into the UK during the 1980s and then spread rapidly through human activities [13,14]. Observations in Spain’s Picos de Europa National Park suggest a recent introduction also and, in both cases, the impacts have been severe [2,15]. Human activities have also been implicated in ranavirus translocations in other parts of the world, for example through amphibian trade, water sports and other behaviors [16–18].

Ranavirus infections have been associated with large numbers of dead and diseased Bosca’s newts (*Lissotriton boscai*) and marbled newts (*Triturus marmoratus*) at a reservoir in Galicia, north-west Spain, since at least 2010 [2]. Ranavirus was also associated with disease and death in a viperine snake (*Natrix maura*), that had been feeding on diseased newts [2]. The reservoir and surrounding area is home to at least 17 species of ectothermic vertebrates [UTM 29T 531504.12m E 4705264.25 m B/ 42°29'44.03"N 8°36'59.99"W, see 19], but very little is known about the overall occurrence, prevalence and impacts of ranavirus in this amphibian and reptile assemblage.

Significantly, in addition to serving as a freshwater source for the surrounding municipalities, the reservoir also serves an important function for agricultural grazing, recreation and sports, as well as national and international canoeing tournaments which may be risk factors for dispersal of viruses known to be persistent in the environment [18,20,21]. This study sought to address the lack of understanding about the local distribution, prevalence and host range of ranavirus at the reservoir and adjacent sites by sampling a broad range of amphibian and reptile species.

## 2. Materials and methods

**Fig 1:**
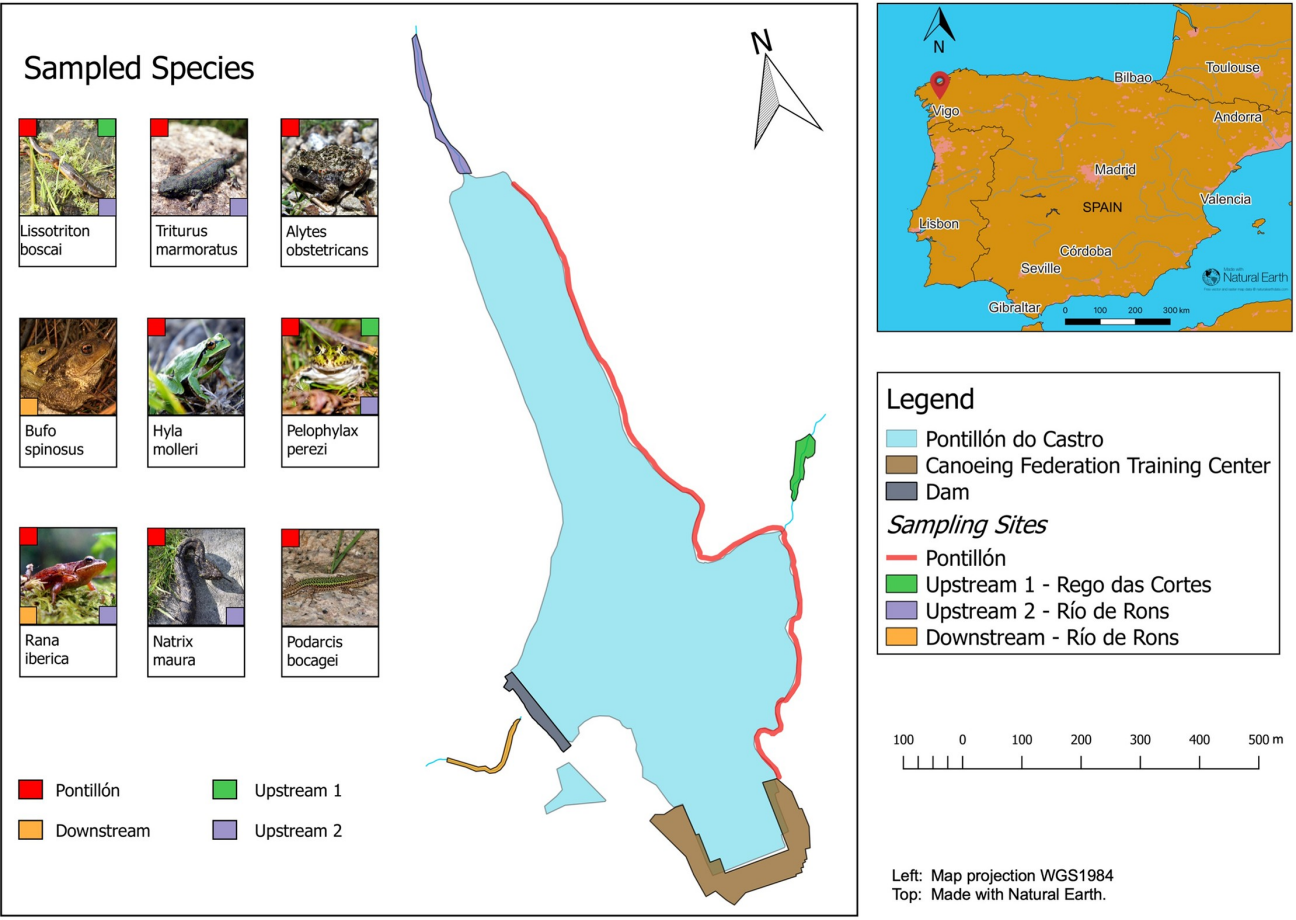
Map of Pontillón do Castro with its tributaries, outlet, sampling sites, and sampled species (42°29'44.03"N 8°36'59.99"W, 213m elevation). The colored squares show the sampling locations for each species.

Fieldwork at Pontillón do Castro reservoir and the surrounding area was conducted in May 2016. All applicable institutional and/or national guidelines for the care and use of animals were followed. Sampling was carried out under the permission of the Galician authorities Xunta Galicia–Consellería de medio ambiente e ordenación do territorio–Dirección Xeral de Conservación da Natureza, permits 420/2015 and 079/2016 for L7R9J1PJZ and 4252/2016 for RX495703. The reservoir is fed by two streams and has one outlet (Fig 1). The eastern banks are shallow and directly accessible, while the northwestern banks are steeper and lack direct access. To assess local prevalence of ranavirus, we employed an area and time-constrained search approach and sampled animals from three types of site: 1) the reservoir itself, along the eastern shoreline, 2) the riverbanks of the tributaries: ‘upstream 1’ and ‘upstream 2’ feeding the reservoir, and 3) the outlet beyond the dam wall: ‘downstream’ (Fig 1).

Surveys of the study sites were performed by using a standardised sampling approach based on the visual encounter surveys (VES) technique (Fig 1). To maximize species diversity of the sample, a range of habitat types were sampled and fieldwork was conducted in all weather conditions and at different times (day and night), following the recommendations Strain et al., 2009 [22]. We sampled more frequently during the day than night, thus increasing the likelihood of encountering diurnal species. The downstream area features difficult terrain and was only visited once when weather conditions were suitable for safe access. Every single individual was recorded only once during our surveys because we were able to keep track of all our movements, and no observer-related effects were expected because the same two observers performed all surveys.

Because there is no single Gold Standard sample collection protocol for ranavirus, we used different techniques for amphibian and reptiles animals as well for dead and alive individuals (see Miller et al. 2015 [25] for a complete review of sampling techniques). Live amphibians—frogs and toads (order *Anura*) and salamanders (order *Caudata*)—were sampled via toe clips or, for small Caudata, via tail clips. Live reptiles were sampled via cloacal swabbing (adults) and buccal swabbing (juveniles) [23–25], using Tubed Sterile Dryswab^TM^ Tip. Tissue biopsies and swab tips were stored in 70% ethanol immediately after collection. After live amphibians were toe- or tail-clipped with fine, dissection scissors, the wound was treated with the antiseptic Bactine^®^ (2.49% Lidocaine Hydrochloride) [26,27] and animals were released at the location they were captured. Dead individuals were collected when available and either stored immediately in 70% ethanol for molecular and pathological examination or frozen at -20°C at the end of each sampling day. Carcasses were swabbed for use in *Batrachochytrium dendrobatidis* diagnostics and dissected to extract liver samples for ranavirus diagnostics. All samples underwent further processing and laboratory diagnostics at the Institute of Zoology (London).

To limit the risk of transmitting infections between individuals or translocating disease agents between sampling locations, nets and scissors were decontaminated between animals using a 1% Virkon^®^ solution (5mg tablets dissolved in 500ml water) for thirty seconds whilst boots were treated with the same solution between sampling locations [28,29].

### 2.1 Laboratory analyses

All DNA extractions were performed using DNeasy^®^ Blood & Tissue kits (Qiagen) following the manufacturers’ instructions. To control for contamination, two negative controls were included in each batch of extractions (extraction reagents minus any sample). Samples were then screened for ranavirus by running duplicate reactions against a standard curve with a hydrolysis probe-based qPCR assay targeting the ranavirus major capsid protein (MCP) gene [30]. Viral quantities were calculated by taking the mean of the viral quantities from the duplicate reactions and analysed using analysis of variance to compare the effects of host species and whether the sample was taken from a live or dead animal.

Ambiguous results (one positive and one negative from duplicate reactions) were rerun, but the non-lethal field sampling methods adopted in this study resulted in a high number of late amplifying samples which were confirmed by a nested PCR targeting a 320 base region of the MCP gene (using Meng *et al*. 2013 [31]). Final primer concentrations were 0.05μM for the first PCR step and 0.4μM for the second PCR step. All reaction mixtures consisted of 4μL of GoTaq^®^ colorless mastermix (Promega), 0.4μL of each primer (forward and reverse), 1.2μL of nuclease-free water, and 2μL of DNA template. Each batch of samples was run with a PCR positive control (extracted DNA from a common midwife toad virus [CMTV] isolate [2]) and a no-template negative control consisting of nuclease-free water. Both PCR steps were run using touchdown PCR settings: an initial 10 minute 95°C step was followed by 23 cycles of 95°C for 30 seconds, 30s at an annealing temperature [initially 62°C and decreasing by 0.5°C with each cycle], and 30s elongation step at 72°C, and a further 25 cycles of 95°C for 30 seconds, 50°C for 30s, and 30s at 72°C. The reaction product from the first PCR was diluted one in ten before use as template in the second PCR step. Products were visualized on a 2% agarose gel alongside a 50bp ladder (Bioline^®^).

A subset of 14 swab samples from fresh carcasses was screened for the presence of *B*. *dendrobatidis* using the method of Boyle et al., 2004 [32]. The single *N*. *maura* carcass was subject to a gross post-mortem examination as it displayed no apparent cause of death.

## 3. Results

We observed seven amphibian and two reptile species over 19 days of field surveys during May 2016 and collected 124 samples across the sampling sites (Table 1). The most frequently sighted species were *L*. *boscai*, *T*. *marmoratus* and the Iberian green frog (*Pelophylax perezi*), occurring in high numbers at the reservoir’s shorelines and the upstream sites.

**Table 1:** Sample numbers and results of Ranavirus (Rv) screening by species and site. The count of carcasses in samples is shown in parentheses. The prevalence by species results combine sampling at all sites for each species.

	Pontillón do Castro		Upstream Site 1 + 2		Downstream		*Total*			
Species	Sample size	Rv positive	Sample size	Rv positive	Sample size	Rv positive	*Sample size*	*Rv positive*	*Prevalence*	*95% Confidence interval*
*Lissotriton boscai*	30 (25)	27 (22)	5 (1)	2 (1)			*35 (26)*	*29 (23)*	*82*.*9 (88*.*5)*	*70*.*4–95*.*3 (76*.*2–101)*
*Triturus marmoratus*	8 (6)	6 (5)	3	2			*11 (6)*	*8 (5)*	*72*.*7 (83*.*3)*	*46*.*4–99 (53*.*5–113)*
*Alytes obstetricans*	2 (2)	2 (2)					*2 (2)*	*2 (2)*	*100 (100)*	*100–100 (100–100)*
*Bufo spinosus*					1 (1)	1 (1)	*1 (1)*	*1 (1)*	*100 (100)*	*100–100 (100–100)*
*Hyla molleri*^a^	18 (0)	11 (0)					*18 (0)*	*11 (0)*	*61*.*1 (na)*	*38*.*6–83*.*6 (na)*
*Rana iberica*^a^	2 (0)	1 (0)	2 (0)	1 (0)	1 (1)	1 (1)	*5 (1)*	*3 (1)*	*60*.*0 (100)*	*17*.*1–103 (100–100)*
*Pelophylax perezi*^a^	5 (2)	4 (2)	36^b^ (1)	15 (1)			*41 (3)*	*19(3)*	*46*.*3 (100)*	*31*.*1–61*.*6 (100–100)*
*Podarcis bocagei*^a^	1 (0)	1 (0)					*1* (0)	*1* (0)	*100* (na)	*100–100(na)*
*Natrix maura*	7 (2)	5 (1)	3 (0)	2 (0)			10 (2)	7 (1)	*70*.*0 (50*.*0)*	*41*.*6–98*.*4 (-19*.*3–119)*
**Total**	**73 (37)**	**57 (32)**	**49 (2)**	**22 (2)**	**2 (2)**	**2 (2)**	**124 (41)**	**81 (36)**	**65.3 (87.80)**	**56.9–73.7 (77.8–97.8)**

^a^ First case of ranavirus detection for this species.

^b^ Ten individuals were captured at Upstream 1, twenty-six at Upstream 2.

**Fig 2:**
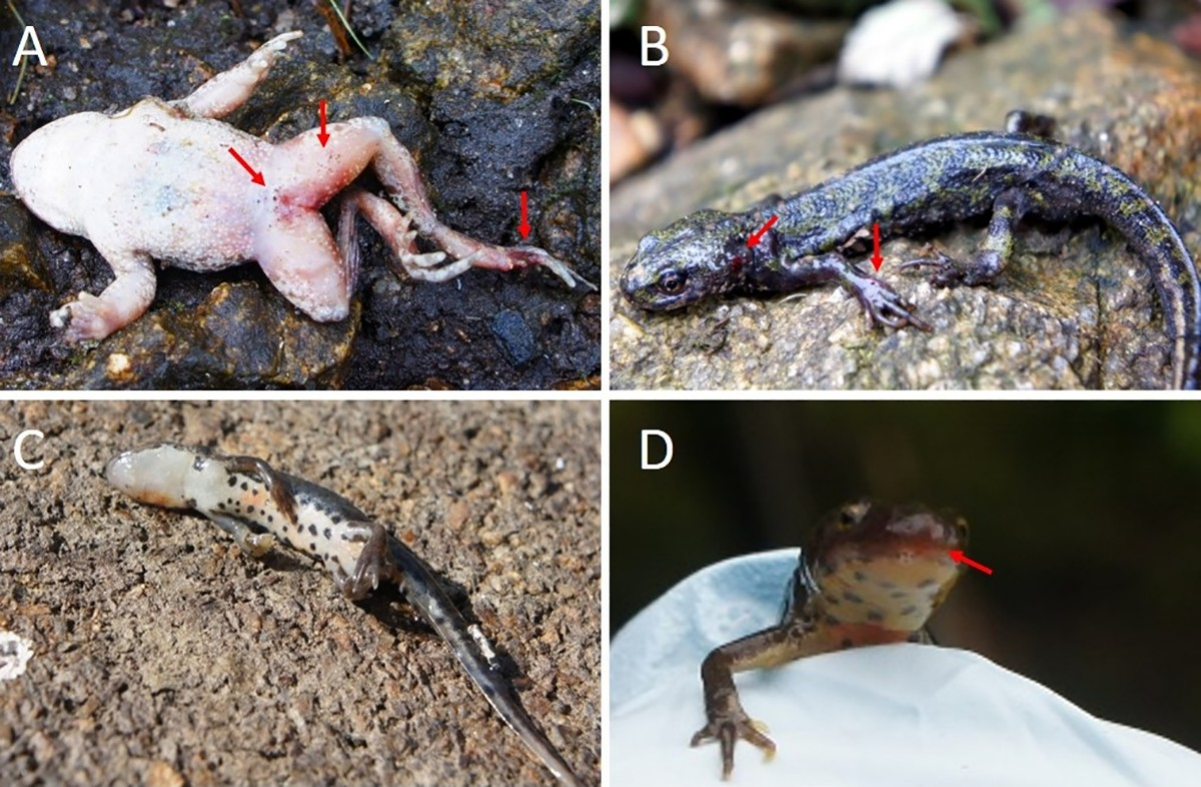
Signs of ranavirosis in three amphibian species. (A) Dead *Alytes obstetricans* with erythema (redness of the skin) around the cloaca and an open lesion on the foot. (B) Live *Triturus marmoratus* with open lesion on the neck and hemorrhaging along digits and the spine, and apparently loose skin. (C) *Dead Lissotriton boscai* without clear outer signs of ranavirosis such as lesions or hemorrhaging. (D) Live *L*. *boscai* with facial hemorrhaging, skin around the mouth appeared swollen and animal seemed to have problems opening its jaws. The species we found dead in most abundance was *L*. *boscai*. Diseased *L*. *boscai* exhibited lethargic, and disoriented behavior. We found apparently diseased *L*. *boscai* within four meters of the edge of the reservoir and seemingly heavily diseased *L*. *boscai* left the water and displayed heavy gasping for breath, behaviors which likely increased detectability of diseased specimens of this species. We did not observe open lesions on *L*. *boscai* which instead presented with subcutaneous hemorrhages and craniofacial swelling (Fig 2D). *Triturus marmoratus* frequently suffered from open skin lesions and lethargic, disorientated movements (Fig 2B). We found *T*. *marmoratus* eggs under terrestrial refuges close to heavily diseased animals (S1 Fig) and several individuals–both live and dead–that were emaciated (loose skin, increased prominence of the spine, Fig 2B).

On each day, we observed numerous dead and dying amphibians presenting with signs indicating ranavirosis. Signs of disease presented broadly as erythema of the skin, hemorrhaging around the mouth, toes, spine, and cloaca, and craniofacial swelling (Fig 2). Animals presenting with external signs of ranavirosis and behavioral changes such as lethargy and uncoordinated movements were found at Pontillón do Castro and upstream site 2. During surveys of the 1.3 kilometer stretch of the eastern shore of the reservoir (Fig 1), we found approximately 10–25 carcasses of *L*. *boscai* and *T*. *marmoratus* per day. We frequently heard the calls of the common midwife toad (*Alytes obstetricans*) at dusk and night time but encountered live animals only during a single night and found two carcasses.

We collected two *A*. *obstetricans* carcasses which had hemorrhages around the cloaca and legs, as well as open lesions (Fig 2A). We did not observe any gross signs of ranavirosis in live or dead specimens of Moller’s tree frog (*H*. *molleri*), Iberian frog (*Rana iberica*), or *P*. *perezi*. Carcasses of *R*. *iberica* and the spiny toad (*B*. *spinosus)* showed signs of predation by Eurasian otters (*Lutra lutra*).

*Natrix maura* was the only snake species observed during this study and Bocage’s wall lizard (*Podarcis bocagei*) the only species of lizard sampled. Neither species displayed physical or behavioral signs of ranavirosis. A single *N*. *maura* carcass was collected exhibiting no visible external signs of ranavirosis. The gross post-mortem found the specimen to be in good physical condition and it was not possible to determine a cause of death.

Quantitative PCR confirmed the presence of ranavirus-infected individuals at all sampling sites. Carcasses accounted for one-third of all samples, thereby introducing a bias towards the presence of ranavirus in the sample set. In total, two-thirds of screened animals tested positive for ranavirus by qPCR (66.9%; n = 124; Table 1). Out of the total 41 carcasses collected, ranavirus DNA was detected in 36 (87.8%). Broken down by sample type, 36/71 toe clips, 2/2 tail clips, 0/1 buccal swabs, 6/7 cloacal swabs, 1/1 skin swabs, 23/25 liver samples, and 13/17 samples from visceral organs were positive for ranavirus. Ranavirus infection was detected in all seven amphibian species sampled, including five species of anuran, *A*. *obstetricans*, *B*. *spinosus*, *H*. *molleri*, *P*. *perezi*, and *R*. *iberica*, as well as two newt species *L*. *boscai* and *T*. *marmoratus*. Both reptile species–*N*. *maura* and *P*. *bocagei*–sampled were also positive.

Of species for which five or more samples were screened for ranavirus, *L*. *boscai* had the highest prevalence (89%; n = 35) and *T*. *marmoratus* had the second highest prevalence (73%; n = 11). Ranavirus was also associated with *H*. *molleri* at high prevalence (61%; n = 18) but we found no carcasses of this species nor did we observe any signs of disease. Viral prevalence was high in *N*. *maura* (70%; n = 10) and *R*. *iberica* (60%; n = 5), and lowest for *P*. *perezi* (46%; n = 41). From the subset of 14 samples for *B*. *dendrobatidis*-screening, 12 *L*. *boscai* samples tested negative, and just one *A*. *obstetricans* out of two tested positive with a load of 0.3 genomic equivalents while both individuals tested positive for ranavirus.

**Fig 3:**
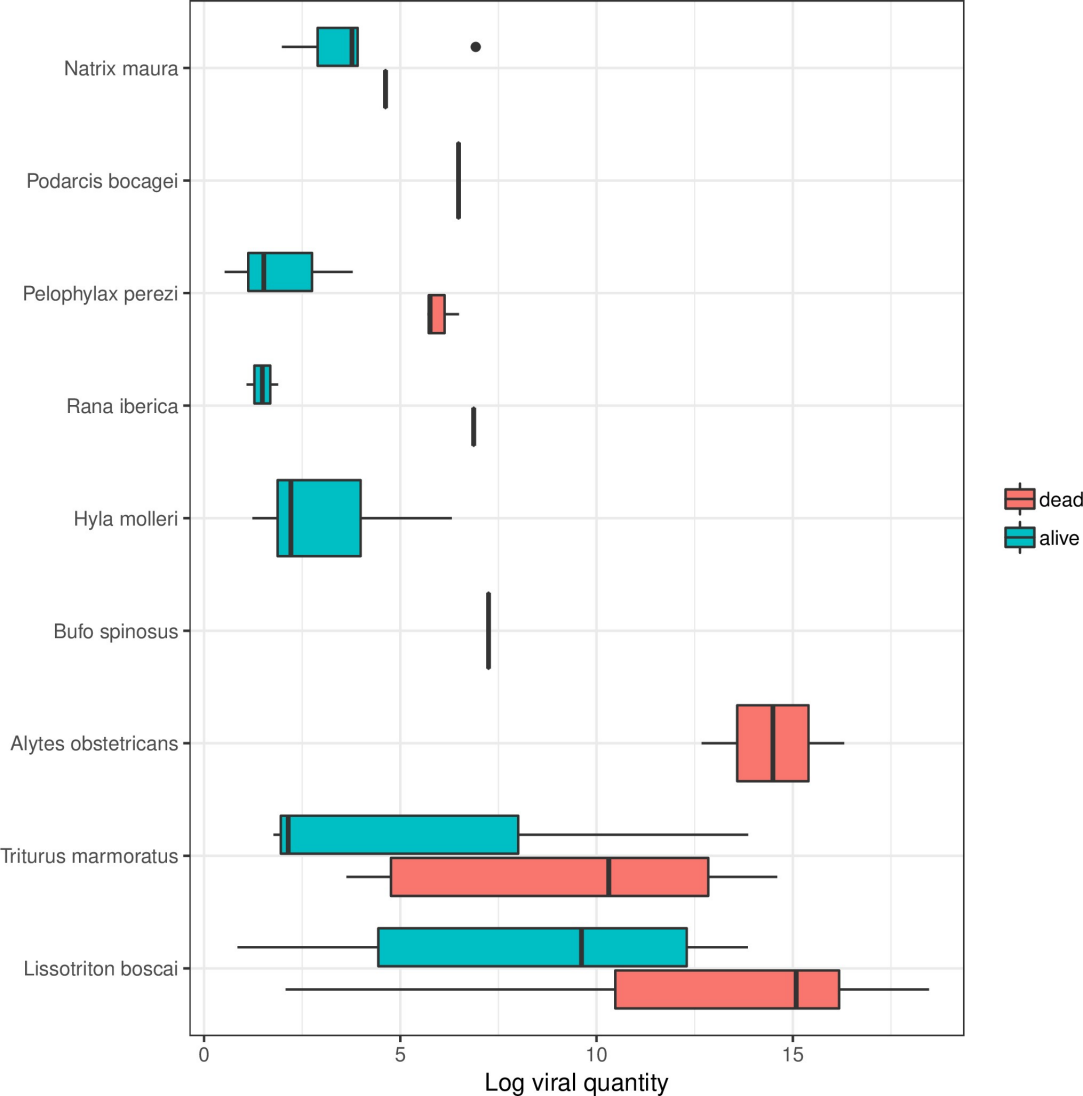
Log viral quantities of infected and uninfected individuals by host and sample type (dead or live specimen). A small increment of 1e-4 was applied to in order to log-transform. Boxplots represent lower quartile, median, upper quartile and interquartile range (upper quartile—lower quartile; central 50% of the data); whiskers extend to the most extreme data point which is no more than 1.5 times the interquartile range from the box; outliers shown as individual points.

Viral quantities were significantly higher in samples from dead animals than those found alive (ANOVA, F_1,114_ = 10.7, p = 0.001) and also varied between species (ANOVA, F_8,114_ = 8.2, p<0.001; Fig 3; S1 Table), with the high quantities found in dead *L*. *boscai*, *A*. *obstetricans* and *T*. *marmoratus* individuals.

## 4. Discussion

Mass-mortality incidents associated with ranavirus infection and disease have affected two caudate species (Bosca’s and marbled newts) at Pontillón do Castro reservoir in north-west Spain since 2010 [2]. Here, ranavirus was detected from samples of an additional seven species and the pathogen was frequently associated with the amphibian and reptile assemblage at the reservoir as well as other sites nearby. This is the first report of ranavirus associated with three frog species (Moller’s tree frog [*H*. *molleri*], Iberian green frog [*P*. *perezi*], and Iberian frog [*R*. *iberica*]) and Bocage’s wall lizard (*P*. *bocagei*). Ranavirus was detected in carcasses of seven species where dead animals were found. However, signs of disease (hemorrhaging/ulceration) were only observed in *L*. *boscai*, *T*. *marmoratus* and *A*. *obstetricans*. The two newt species were the most severely affected species based on the number of carcasses found.

Individuals from several species with subclinical ranavirus infections were found. Ranavirus was frequently detected in *P*. *perezi* and *H*. *molleri* but no signs of ranavirosis were observed in any individuals of these species. It is possible that mortality went undetected in these species given the difficulties in sampling a large waterbody of this type, particularly if larval or metamorphic stages are more susceptible as commonly found in other species and systems, or because of the timing of our sampling only covered only part of the season. Controlled laboratory exposure experiments would be necessary to assess the relative susceptibility of species in this community. The high diversity of amphibians and reptiles at Pontillón do Castro and the variation in outcomes following exposure increases the likelihood of ranavirus persistence [33,34].

In light of the dramatic effects of CMTV in the nearby Picos de Europa National Park [2], the presence of a ranavirus in Pontillón do Castro may threaten the stability of susceptible ectothermic vertebrate communities. A previous study reported recurring mortality in newts [2] and the Spanish Herpetological Society (AHE) conducted carcass counts at Pontillón do Castro (31 days between 2010 and 2015) and found 1,796 carcasses of *L*. *boscai* and *T*. *marmoratus* (AHE, unpublished data). During fieldwork for this study, between ten and 25 carcasses were observed daily, confirming this trend for 2016. Our sampling effort was limited to May 2016 and the setting of Pontillón do Castro makes monitoring population counts very challenging, but such a high level of persistent mortality is a concern, particularly in the context of the drastic impacts of ranavirus on herpetofauna observed elsewhere [2,15,35].

Our observations at Pontillón do Castro are in line with previous reports of high infection prevalence and sudden mortality in multiple species [25]. Prevalence levels in all sampled amphibian species are above suggested background prevalence levels for amphibians [36] and laboratory experiments have shown strong correlation between infection prevalence and mortality [34]. We frequently observed mortality in *L*. *boscai* and *T*. *marmoratus* for which we reported prevalence levels of 82.9% and 72.7% respectively. For *H*. *molleri* and *P*. *perezi* we observed high prevalence (60% and 46.3% respectively) but no or little mortality or signs of disease. While this might be due to behavioral patterns of the species or limitations in sampling that preventing us from finding more carcasses, high ranavirus-prevalence with no signs of ranaviral disease have been reported in other species [37,38]. Further, it has been reported that anthropogenic disturbance and presence of cattle correlate with high ranavirus prevalence [39–41], both of which occur at Pontillón do Castro. Note, however, that these values of prevalence should be treated with caution because tail/toe-clip sampling can underestimate the true prevalence of ranavirus in wild amphibian populations [42] and, on the other hand, and due to the large number of dead animals at our study sites, surface contamination could has happened.

Our study provides valuable new insights on the effect of ranavirus on snakes in the wild. The Iberian grass snake, *Natrix astreptophora* [formerly *N*. *natrix*, see 43] has been reported to hunt and scavenge along the shores of Pontillón do Castro [44] but no individuals were detected during this study. However, we did record high ranavirus prevalence in the related *N*. *maura* (70%), extending previous observations of disease and mortality in this species [2]. These findings highlight the possible negative impacts of ranavirus on *Natrix* populations and need for further investigation as a priority.

Pontillón do Castro is part of a network of streams and waterbodies. We found ranavirus to be present in both upstream sites suggesting that it is not restricted to still waters but can persist where hosts occupy smaller, flowing waters. This represents a potential means of pathogen dispersal in addition to terrestrial amphibian dispersal into neighboring waterbodies [20,23,45,46]. Another potential route of transmission is through incidental transportation of contaminated sediment by humans or animals both frequently present at Pontillón do Castro [45,47]. The reservoir is used for mountain-biking, angling, dog-walking, and watersports. Since 2007, the Galician Canoeing Federation hosts tournaments at Pontillón do Castro with national and international participants, thus increasing the risk for ranavirus dispersal through boats and equipment [18,48]. There is no biosecurity protocol in place, further increasing dispersal risk through water or soil trapped in equipment, clothing, or vessels.

The wide host range of ranaviruses, the possibility of anthropogenic and natural dispersal, and the interconnectedness of Pontillón do Castro pose a risk of infection for susceptible species in the region and potentially across the Iberian Peninsula. The uncertainties around the modes of ranavirus transmission and dispersal, its persistence in the environment as well as the impact of outbreaks for specific host communities highlight the importance of biosecurity measures and educational programs to prevent further dispersal and to raise awareness among the general public and the stakeholders of Pontillón do Castro.

## Supporting information

S1 Fig (JPG)

S1 Table (DOCX)
