# Supplementary figures and images for: High pathogen prevalence in an amphibian and reptile assemblage at a site with risk factors for dispersal in Galicia, Spain
Source: PLoS One. 2020 Jul 30;15(7):e0236803. doi: 10.1371/journal.pone.0236803 (PMC7392302; doi:10.1371/journal.pone.0236803)

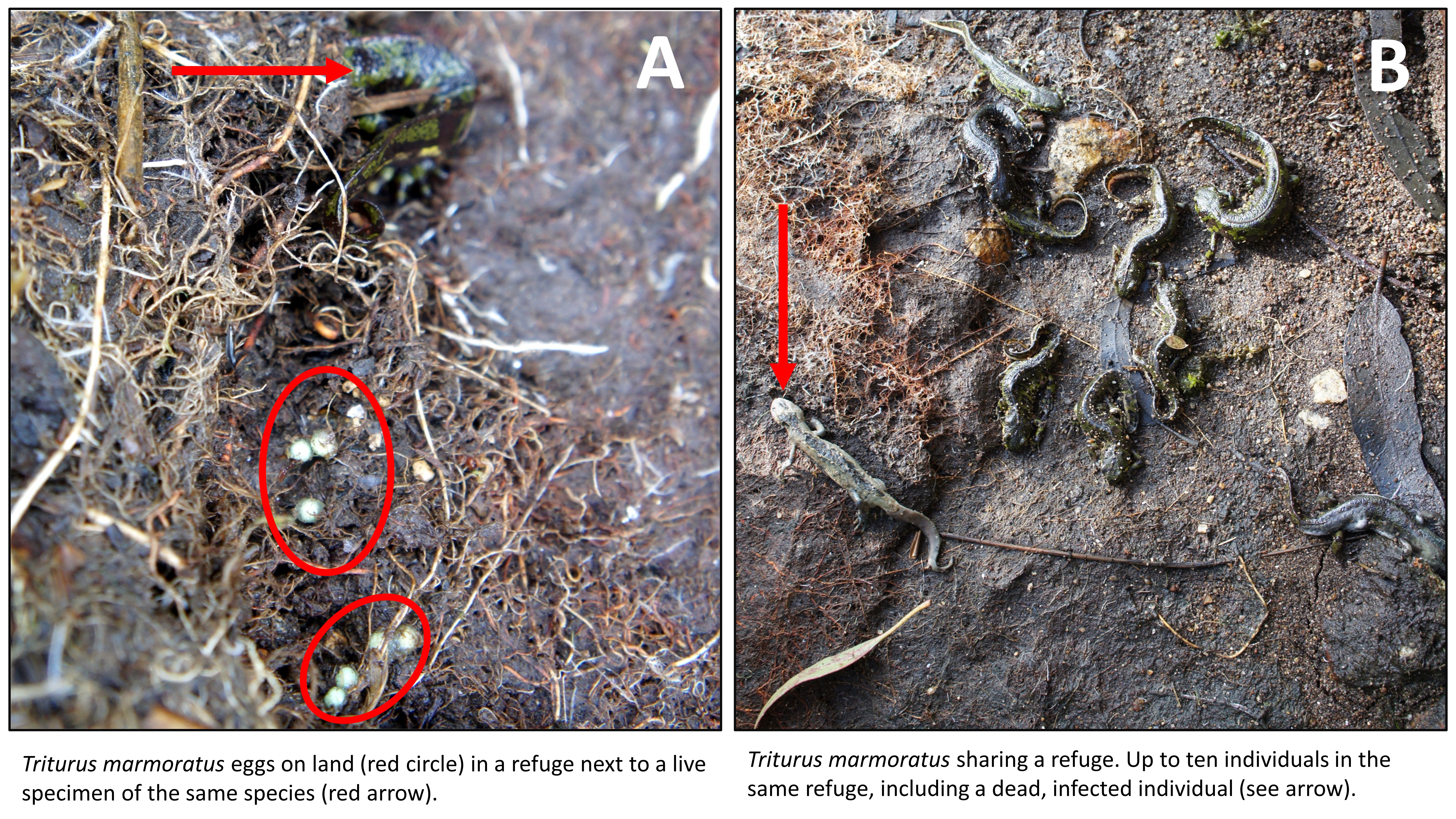

Supplement: S1 Fig — (JPG) [file pone.0236803.s001.jpg]
