# Supplementary material for: High pathogen prevalence in an amphibian and reptile assemblage at a site with risk factors for dispersal in Galicia, Spain
Source: PLoS One. 2020 Jul 30;15(7):e0236803. doi: 10.1371/journal.pone.0236803 (PMC7392302; doi:10.1371/journal.pone.0236803)
Supplement: S1 Table — (DOCX) [file pone.0236803.s002.docx]

**Table S1. Viral loads and standard deviations by species and sample source.**

| **Species** | **Sample source** | **Count** | **Mean viral load** | **Standard deviation** |
| --- | --- | --- | --- | --- |
| *Lissotriton boscai* | Live specimen | 6 | 246,000 | 404,000 |
|  | Carcass | 23 | 10,400,000 | 22,200,000 |
|  | Total | 29 | 8,330,000 | 20,100,000 |
| *Triturus marmoratus* | Live specimen | 3 | 350,000 | 606,000 |
|  | Carcass | 5 | 521,000 | 950,000 |
|  | Total | 8 | 457,000 | 793,000 |
| *Alytes obstetricans* | Carcass | 2 | 6,210,000 | 8,330,000 |
| *Bufo spinosus* | Carcass | 1 | 1,400 | NA |
| *Hyla molleri* | Live specimen | 11 | 74.10 | 161.00 |
| *Rana iberica* | Live specimen | 2 | 4.78 | 2.58 |
|  | Carcass | 1 | 963 | NA |
|  | Total | 3 | 324 | 553 |
| *Pelophylax perezi* | Live specimen | 16 | 10.70 | 12.40 |
|  | Carcass | 3 | 425 | 206 |
|  | Total | 19 | 76 | 170 |
| *Podarcis bocagei* | Live specimen | 1 | 654 | NA |
| *Natrix maura* | Live specimen | 6 | 196 | 402 |
|  | Carcass | 1 | 102 | NA |
|  | Total | 7 | 182 | 369 |
